# Occupational risk of cutaneous larva migrans: A case report and a systematic literature review

**DOI:** 10.1371/journal.pntd.0010330

**Published:** 2022-05-12

**Authors:** Angela Stufano, Caterina Foti, Piero Lovreglio, Paolo Romita, Aurora De Marco, Riccardo Paolo Lia, Domenico Otranto, Roberta Iatta

**Affiliations:** 1 Interdisciplinary Department of Medicine, University of Bari, Bari, Italy; 2 Department of Biomedical Science and Human Oncology, Dermatological Clinic, University of Bari, Bari, Italy; 3 Department of Veterinary Medicine, University of Bari, Bari, Italy; 4 Faculty of Veterinary Sciences, Bu-Ali Sina University, Hamedan, Iran; International Atomic Energy Agency, AUSTRIA

## Abstract

Cutaneous larva migrans (CLM) is a parasitic zoonosis of warm tropical and subtropical areas, although autochthonous cases have been increasingly reported in Western European countries. Data on the prevalence of CLM as an occupational disease in workers exposed to potentially contaminated soil or in close contact with dogs and cats are scant. Herein, we report an autochthonous case of CLM in a dog breeder from southern Italy (Apulia region), along with a systematic literature review describing the risk of CLM infection, mainly according to job categories. The patient was referred to the dermatology unit presenting a serpiginous lesion on his hand, raising the suspected CLM diagnosis. In non-endemic areas, CLM might represent a challenge for physicians in terms of diagnosis, treatment, and prevention, particularly in workplaces. The multidisciplinary approach in the diagnosis of CLM with the involvement of different scientific competences (i.e., dermatologists, veterinarians, and occupational physicians) may contribute to further assess the distribution of human CLM and associated risk factors, toward reducing the risk for the infection.

## Author summary

Cutaneous larva migrans (CLM) is a parasitic zoonosis caused by multiple types of hookworms, characterized by creeping migration of larva through the skin. Humans are infected through accidental penetration of their skin by free-living larvae at the filariform stage. CLM is typically acquired in warm tropical or subtropical areas, although reports of autochthonous cases have increased in Western European countries. There is little evidence on the prevalence of CLM as an occupational disease, although workers exposed to potentially contaminated soil or to animals that are the definitive hosts of these nematodes, as dogs and cats, could be considered at higher risk of infection. We report a case of autochthonous CLM in a dog breeder in southern Italy (Apulia) to highlight how the collaboration among specialists in a One Health perspective (e.g., dermatologists, occupational physicians, veterinarians, and parasitologists) is essential for the correct diagnosis of zoonotic infection.

## Introduction

Cutaneous larva migrans (CLM) is a parasitic zoonotic disease primarily caused by larval skin migration of soil-transmitted hookworms (Ancylostomatidae). The most frequent causative agents of human CLM infest the gastrointestinal tract of the definitive hosts such as dogs and cats (i.e., *Ancylostoma braziliense*, *Ancylostoma caninum*, and *Uncinaria stenocephala*) and cattle (i.e., *Bunostomum phlebotomum*), therefore causing cutaneous creeping lesions while penetrate into the skin of accidental host from the environment [1]. Similarly, the human intestinal hookworms (i.e., *Ancylostoma ceylanicum*, *Ancylostoma duodenale* and *Necator americanus*) penetrate percutaneously and cause a local pruritic, erythematous, papular rash mimicking a cutaneous eruption [1].

While CLM disease is considered endemic in warm tropical and subtropical areas, reports of autochthonous cases have been increasing in European countries (e.g., Germany, England, France, Italy, Spain, and Serbia) [2–7]. The adult Ancylostomatidae parasites reside in the small intestine of the definitive hosts (i.e., cattle, dogs, and cats) with eggs shed through the feces into the environment [8]. Following human infection by accidental larval penetration in the skin, small pruritic erythematous papules or vesicles occur with the formation of creeping paths through the corneal layers of epidermis [9]. Larvae generally advance at a rate of about 1 to 3 cm per day and produce skin rashes that evolve with a peculiar serpiginous aspect [1,10]. The diagnosis is usually based only on the clinical examination, since laboratory findings may be unremarkable and/or not specific [11]. Therefore, delayed or misdiagnosis are possible events, resulting in inappropriate treatments [12]. Although larvae cannot penetrate the skin basal membrane leading to a spontaneous resolution in about 1 to 2 months, secondary complications may occur (e.g., local or general allergic reactions, secondary bacterial infections by *Staphylococcus aureus* and *Streptococcus* species, Lӧffler syndrome, and eosinophilic enteritis), suggesting the importance of a prompt etiological diagnosis [13,14].

Under the above circumstances, workers exposed to animals (i.e., dogs and cats), which potentially harbor ancylostomatids, and workers in contact with potential contaminated soil or sand, such as breeders, farmers, agriculturists, gardeners, even in absence of animal contact, may be considered at high risk of infection, therefore suggesting the risk of CLM as an occupational disease. Here, we report an autochthonous case of CLM in a dog breeder in southern Italy along with a systematic literature review describing the risk of CLM disease mainly in job categories in non-endemic areas.

### Case report

**Fig 1:**
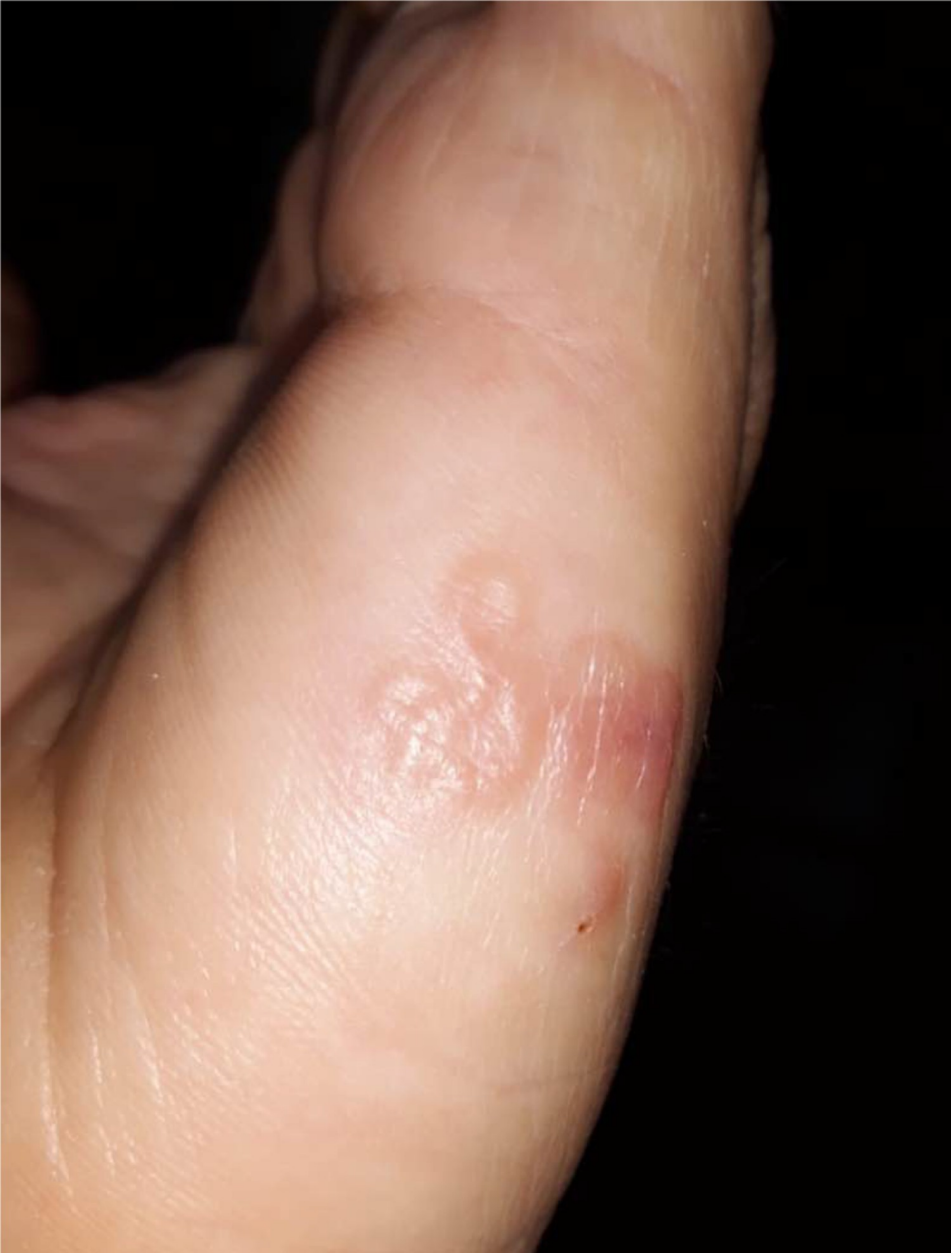
Skin lesion on left hand of the patient at the clinical examination at the dermatology ward.

**Fig 2:**
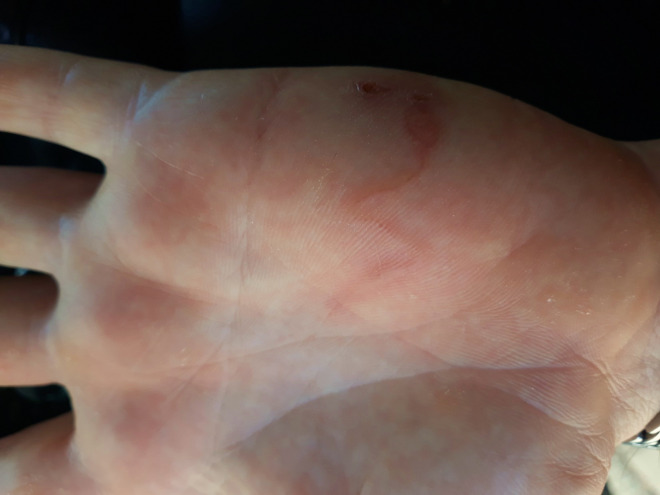
Skin lesion on left hand of the patient 6 days later the first clinical examination at the dermatology ward.

In September 2020, a 48-year-old male patient was referred to the dermatology ward of University Hospital of Bari (Italy) for a serpiginous cutaneous track that had appeared 10 days before as a point-like crusted element, on the internal side of his left hand (Fig 1). The patient referred that the lesion had already been topically treated with acyclovir cream twice daily for 5 days as an herpetical infection was suspected by the general practitioner. Since then, the lesions had advanced by l to 2 cm per day (Fig 2), with papules at the starting point. No signs of dermographism, burrows, or lymphadenopathy were observed.

The patient did not refer any previous significant disease and any travel abroad Italy in the past 2 years, but reported to work as a dog breeder of American Staffordshire Terrier since 20 years. Actually, he referred to breed a bitch and five 1-month old puppies, but did not mention any recent dog bites or scratches. In addition, the patient referred to handle animals by bare hands while cleaning the shelter, feeding the dogs, administering endoparasiticides, and walking the dogs.

Routine blood tests were performed resulting normal and particularly eosinophilia was not observed. Based on the peculiar creeping aspect of the cutaneous lesion, a skin scraping was performed from the patient palmar left hand of about 1 cm beyond the lesion, along with serum sample collection for serological testing.

Genomic DNA was extracted from the skin by using a commercial kit (DNeasy Blood & Tissue Kit, Qiagen, Hilden, Germany) and analyzed by conventional PCR using specific primers targeting a portion of cytochrome c oxidase subunit 1 (*cox*1) gene for nematodes’ DNA [15]. No nematode’s DNA, including *Strongyloides stercoralis*, *Dirofilaria immitis*, and *Dirofilaria repens* or belonging to Ancylostomatidae family, was detected. The serum sample tested for the detection of IgG against *D*. *repens* and *D*. *immitis* by an enzyme-linked immunosorbent assay resulting negative [16].

Following a discussion of the case report with colleagues of parasitology unit of the Department of Veterinary Medicine at the same University, a One Health approach was advised, and 3 fecal samples were collected from the bitch bred by the patient. Samples were analyzed by coprological tests (i.e., direct microscopy, Baermann, and flotation methods) for the detection of intestinal parasites. Briefly, for the Baermann examination, 5 grams of feces were analyzed, and after 18 hours, the sediment was microscopically observed, whereas a fecal flotation was performed on 2 g of feces by using a zinc sulfate solution (ZnSO_4_) with a specific gravity of 1.35 [17]. The eggs isolated were measured by an optical DM-LB2 microscope and Leica LAS version 4.5.0 software (Leica Microsystems, Wetzlar, Germany) and identified according to morphological keys [17].

Genomic DNA was extracted from single eggs isolated by flotation method and preserved in 70% ethanol, using a commercial kit (QIAamp, DNA Micro Kit, Qiagen) in accordance with the manufacturer’s instruction. A conventional PCR targeting the 18S rRNA gene was performed, according to the protocol by Patterson-Kane and colleagues [18], and the amplicons were purified and sequenced using the Taq Dye Doxy Terminator Cycle Sequencing Kit (v.2, Applied Biosystems, Foster City, California, United States of America) in an automated sequencer (ABI-PRISM 377). Sequences were compared with those available in the GenBank database by Basic Local Alignment Search Tool (BLASTn, http://blast.ncbi.nlm.nih.gov/Blast.cgi).

**Fig 3:**
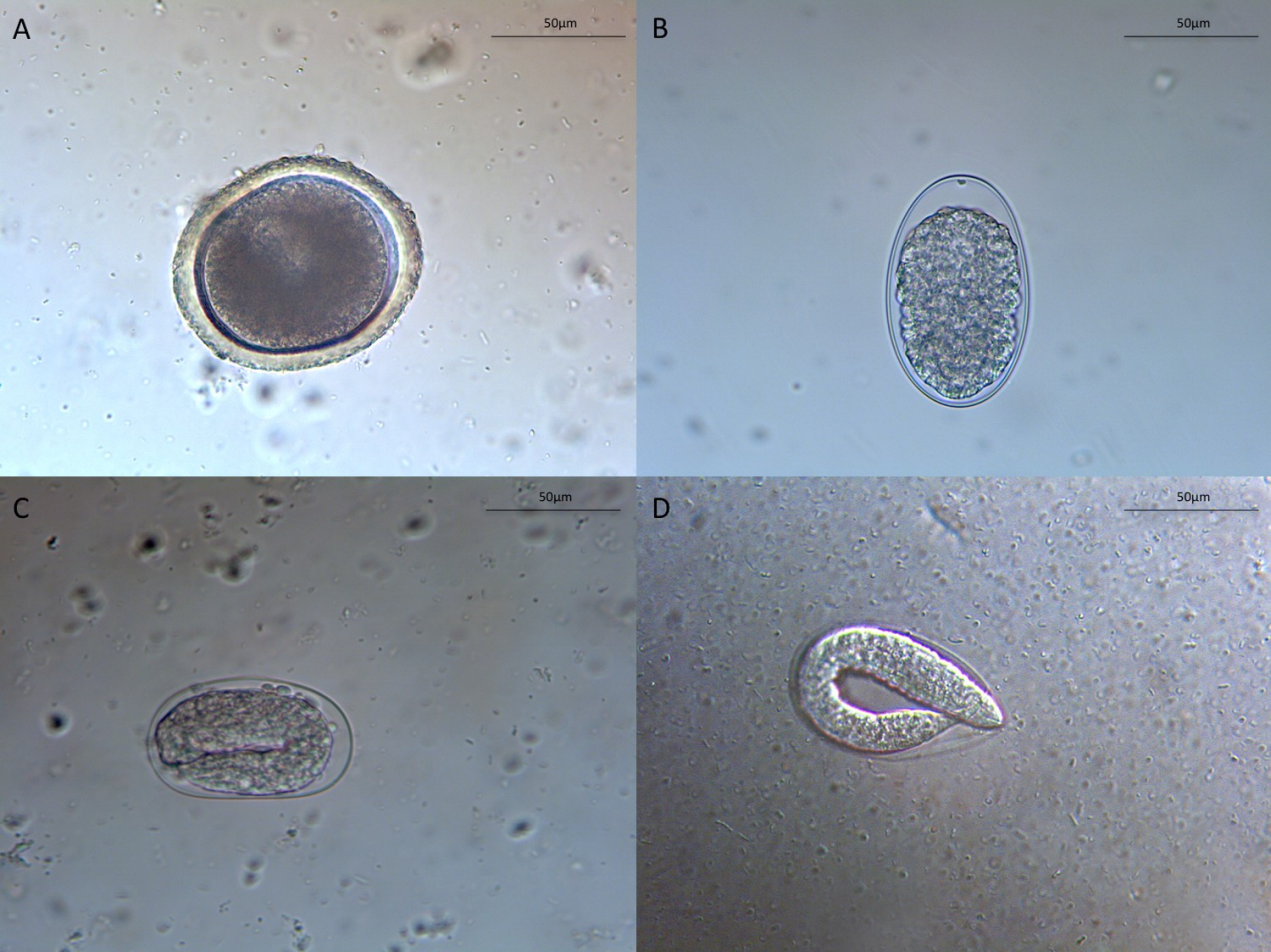
Morphology of *Toxocara canis* egg **(A)**; morphology of unembryonated **(B)**, embryonated **(C)** and with first-stage larva eggs of *Ancylostoma caninum* **(D)**.

Intestinal nematode eggs of 2 different genera were isolated by flotation from the dog feces and identified as belonging to *Toxocara canis* (Fig 3A) and Ancylostomatidae family (Fig 3B–3D). These latter eggs were analyzed by PCR and identified by BLAST analysis of the partial 18S rRNA gene sequence as *A*. *caninum* displaying 99.3% nucleotide identity with that recovered from a domestic dog (GenBank accession number AJ920347). No first-stage larvae of *S*. *stercoralis* were detected at the Baermann test. The patient was treated with systemic albendazole (400 mg for 3 days), the itch stopped in 1 day and, after 3 days, cutaneous lesions was completely recovered confirming the suspicion of the parasitic cutaneous infection.

After a preliminary evaluation by the Internal Board on the medical procedures to be applied, formal written informed consent was previously obtained from the patient for the publication of his case details.

## Methods

**Fig 4:**
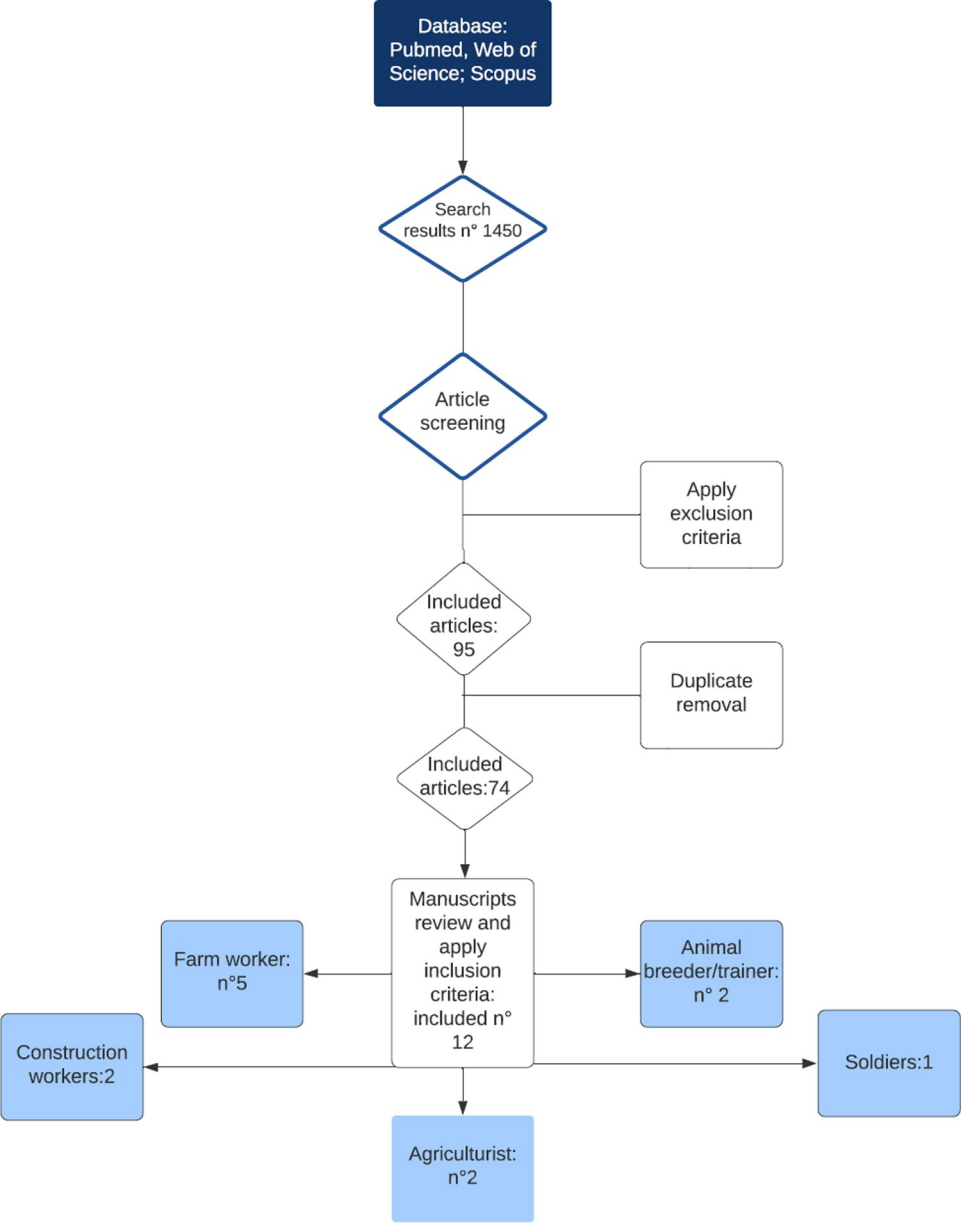
Description of the selection process for articles included in the review (PRISMA method). PRISMA, Preferred Reporting Items for Systematic Reviews and Meta-Analyses.

A systematic literature search on the possible occupational source of CLM has been herein conducted on January 2022, by sourcing both National Library of Medicine (NLM) resources through PubMed, and Scopus and Web of Science, using the following keywords: (“Cutaneous Larva Migrans”) AND (“worker” OR “job” OR “occupational” OR “case report” OR “farmer” OR “agriculturist” OR “breeder”). The screening of resources took place in the first phase by reading the articles’ titles and abstracts and removing the duplicates, resulting in the selection of 74 items (Fig 4). In the second stage, the articles were analyzed according to the inclusion criteria, as English language studies, case reports or research articles, topic on CLM in occupational setting, and exclusion criteria, as non-occupational setting studies or other nematodes studies and non-cutaneous manifestations of infections or studies regarding seroprevalence rates without clinical manifestations, resulting in the selection of 12 items. The article details included in the review are reported in Table 1, according to the job performed. In addition, the Ottawa criteria for a good assessment were considered in assessing the quality of manuscripts [19].

**Table 1:** Studies on occupational CLM from 1965 to 2021.

Study	Country	Case study	Job	Diagnosis	Body site of the lesion
Honeycutt and colleagues (1965) [22]	Arkansas, USA	12 workers of building trades	Construction employees	Clinical examination; negative blood count, sputum and stool examination, skin biopsy	Multiple larva penetration in 8 cases, localized in 4 cases
Belsole and colleagues 1980 [23]	Florida, USA	50-year-old male	Dog breeder	Clinical examination	Dorsum of the hand and index finger
Green and colleagues (2001) [24]	Belize (1999)	13 out of 15 British soldiers infected	2-week jungle training exercise	Clinical examination	No. 12 on legs, no. 2 on foot or ankle
Kim and colleagues (2006) [25]	South Korea (2003)	Two 74- and 62-year-old males	Farm workers	Clinical examination; negative blood test	Right ankle and calf, left foot
Conde and colleagues (2007) [26]	USA	38-year-old, male, Guatemalan (USA in the past 8 years)	Farm worker	Clinical examination	Dorsal surface right foot
Upendra and colleagues (2013) [27]	India	50-year-old female	Agriculturist	Clinical examination; skin biopsy, negative blood tests	Left wrist, index and middle fingers
Akkouche and colleagues (2015) [10]	Italy	42-year-old, male	Livestock technician (intensive farms)	Clinical examination; negative stool, skin biopsy, blood tests	Wrist right forearm up the arm to the right shoulder; smaller size lesion left forearm
Kokollari and colleagues (2015) [28]	Kosovo	37-year-old male	Farm worker	Clinical examination; negative blood test	Gluteus region
Nurjahan and colleagues (2016) [29]	Malaysia	40-year-old, foreign, male	Agriculture worker (palm oil plantation)	Clinical examination	Abdomen
Rashid and colleagues (2017) [30]	UK	47-year-old male	Guide dog trainer	Clinical examination	Right hand
Sharma and colleagues (2017) [31]	India	65-year-old male	Farm worker	Clinical examination; skin biopsy	Abdomen and chest
Sil and colleagues (2021) [32]	India	40-year-old male	Hod carrier	Clinical examination; dermoscopy	Scalp

CLM, cutaneous larva migrans.

## Discussion and review of the literature

CLM is usually referred as a tropical disease and mainly described in returning travelers from endemic areas such as Africa, Southeast Asia, and South America [20]. In the latter cases, a detailed travel and medical history of patients living in non-endemic areas are fundamental for reaching a correct diagnosis and a prompt treatment [21].

In our literature review, 3 case reports were probably related to occupational contact with animals, 6 to agricultural and farm setting, one to a group of military workers and the last 2 to construction workers infected by direct contact with potentially contaminated soil or sand (Table 1) [10,22–32]. Out of 12 reviewed cases, 7 were reported in patients living in non-endemic areas (i.e., the United Kingdom, Italy, Kosovo, South Korea, and the USA), suggesting a wide distribution of CLM. CLM-associated nematodes of veterinary concern in Western areas are *A*. *caninum* and *U*. *stenocephala* being the latter well adapted to temperate climates and widespread in red fox (*Vulpes vulpes*) population and in free-ranging gray wolves (*Canis lupus*) in Italy [33].

CLM is a parasitic zoonosis endemic in geographical areas characterized by warm and humid climate favoring the environmental circulation of helminths. The global warming and therefore the tropicalization of European climate may promote the emergence of tropical parasites such as *A*. *braziliense* detected in patients from southern Europe (i.e., Italy, Spain, and France) [34].

In this scenario, job categories, such as those in close contact with domestic animals or soil contaminated by animal feces, are potentially exposed to CLM infection. This is confirmed by the case report herein described from a dog breeder, along with those reported in the literature (Table 1) specifically including a livestock technician and 2 dog trainer/breeder [10,30], all presenting creeping eruption in the hands. Therefore, handling animal fecal samples or contaminated soil by workers without any protective measure may represent a source of infection also in countries of the northern hemisphere [12]. Another example on the circulation of geohelminths, such as *T*. *canis* and *A*. *caninum*, through dog fecal contamination in urban area, reported in the same geographical area of the CLM case herein described, highlighted the importance of dogs in shedding these parasites of zoonotic concern thus representing an infection risk for humans living in non-endemic areas [35].

Other anatomical sites of CLM may be the foot, abdomen, and chest as reported in farmers, agriculturists, and construction workers also as a consequence of exposure to physical or chemical agents that may contribute to skin irritation and larva penetration [22–27,29,31]. However, even if the lesions are usually observed on feet and legs, they can occur on any body site in direct contact with contaminated and wet soil or sand, as showed for a hod carrier ferrying sandbags at a construction site, reporting a scalp serpiginous eruption [32]. In addition, in agricultural setting, uncommon clinical presentation can occur such as hair follicle inflammation (hookworm folliculitis), most frequently in the gluteal region, probably because larvae may be transferred to human skin through contaminated clothes, towel or other objects [11]. In farm setting, moreover, co-exposure to different biological agents may evoke contextual inflammatory and/or allergic reactions that could facilitate secondary bacterial infection resulting in atypical clinical manifestations of CLM [28].

The exposure of bare skin to water and mud heavily contaminated with viable larvae is also reported in a troupe (*n* = 13) of British military showing serpiginous lesions in their legs after their training period in the jungle, despite the use of protective trousers [24]. Indeed, hookworm larvae are excellent swimmers and can survive for weeks in humid environment [1]. In this case, extremely contaminated soil in a hyperendemic area, such as Belize, may have allowed larvae to penetrate through porous trousers and soil within the boots [36]. Therefore, these data suggest the importance of training workers in using appropriate clothing and personal protective equipment during working activities when in contact with animals or potentially contaminated soil.

Different occupational risk factors may contribute to an increased incidence of CLM in workplaces. First, handling compost or using stool as fertilizer represent an important source of infection for farm and agricultural workers. In addition, cutaneous lesions caused by injury from agricultural tools or scratches and wound related to animal contact could promote larva penetration through the skin. Finally, in rural areas with high prevalence of CLM, poverty, low education along with poor hygiene conditions, and limited access to safe water could contributed to a higher risk of infection in occupational categories [37]. As far as for construction workers, tools and materials used for dwellings represented a source of infection for soil-transmitted helminths (STHs) [38].

Following the “One Health” approach, the suspected CLM infection in the dog breeder herein diagnosed was arisen through the direct detection of the parasite in dog’s feces also in absence of any molecular positive result in skin samples of the patient. In this case, the fast migration of larvae, as well as the superficial skin sampling, may have reduced the chances for the detection of parasite DNA. Although the lack of the etiological diagnosis may be considered a limitation of the study, nevertheless, the detection of zoonotic parasite in dog fecal sample allows to exclude other possible causes of skin manifestations with similar clinical pictures and potentially associated with occupational exposure to contaminated soil or infected animal.

The differential diagnosis for occupational CLM could include scabies, *tinea corporis* by dermatophytes, contact dermatitis, erythema chronicum migrans, gnathostomiasis, loiasis and phytophotodermatitis [19]. In addition, creeping skin eruption may be due to *larva currens* caused by *S*. *stercoralis*, a parasite of zoonotic concern, mainly shed through feces by dogs representing the main reservoir [39]. In the latter case, the penetration of the third-stage larvae causes a mostly chronic infestation, with mild or severe symptoms according to the dissemination in human body and the specific host immune reactiveness [40]. In addition, gnathostomiasis, paragonimiasis, and fascioliasis may also cause cutaneous lesions indicating that anamnestic data and knowledge of the epidemiology of these diseases should be truly considered while diagnosing skin conditions [41]. Overall, awareness about animal diseases present in specific contexts is pivotal to perform the occupational risk assessment and to adopt as well as implement targeted prevention strategies in the workplace. Based on this evidence, a One Health approach may help the risk assessment of occupational CLM infection, since climatic conditions, soil characteristics as well as the presence of animals acting as reservoirs, are essential factors for the geohelminth occurrence [42]. In addition, human activities, such as fragmentation of the environment, and land use, as well as deforestation and urbanization, ultimately cause changes in ecosystems and rural landscapes into peri-urban areas, leading to increase of wildlife as demonstrated for zoonotic parasites of reptiles and carnivores, mainly when considering vector-borne diseases [33,43–45].

## Conclusions

In non-endemic areas, CLM might represent a challenge for physicians in terms of diagnosis, treatment, and prevention, particularly in workplaces where it could be considered a neglected disease. Improvement of sanitation, health surveillance programs, and training dedicated to a better knowledge of the disease and its associated risk factors should be considered the essential strategies to be implemented in high-risk occupational settings. In a One Health perspective, the collaboration among specialists (e.g. dermatologists, occupational physicians, veterinarians, and parasitologists) is advised for a correct diagnosis of zoonotic disease such as CLM and for adopting correct preventative measures for reducing the risk of infection in particular job categories.

**Key learning points**

- Multidisciplinary approach is essential for the diagnosis of cutaneous larva migrans (CLM).
- In workplaces, CLM could be considered a neglected disease.
- Correct preventative measures should be applied for reducing the infection risk in specific job categories.
